# Evidence-based management of chemotherapy-induced nausea and vomiting: a position statement from a European cancer nursing forum

**DOI:** 10.3332/ecancer.2011.211

**Published:** 2011-04-28

**Authors:** C Vidall, P Dielenseger, C Farrell, E Lennan, P Muxagata, P Fernández-Ortega, K Paradies

**Affiliations:** 1 Clinical Risk and Practice Development, Healthcare at Home, Bristol, UK; 2 Clinical Research and Early Clinical Trials Unit, Institut Gustave-Roussy, Paris, France; 3 The Christie Hospital, Manchester, UK; 4 Southampton University Hospitals Trust, Southampton, UK; 5 Oncology Unit, Sousa Martins Hospital, ULS Guarda, EPE, Portugal; 6 Nursing Research, Institut Català d’Oncologia, L’Hospitalet, Barcelona, Spain; 7 Gynaecological and Oncological Outpatient Clinic Prof Dr Schmidt Rohde, Hamburg, Germany

## Abstract

Chemotherapy-induced nausea and vomiting (CINV) is a common, but now often overlooked side effect of cancer treatment, and one that can be largely prevented through the implementation of international evidence-based guidelines. The European CINV Forum, comprising nurses from France, Germany, Portugal, Spain and the UK, discussed the use of CINV preventive strategies in routine practice, and the factors that affect optimal delivery of antiemetic therapies. Based on these discussions, they developed a series of recommendations for optimal, evidence-based management of CINV. These state that all patients receiving chemotherapy should undergo full assessment of their risk of CINV and receive appropriate prophylactic treatment based on guidelines from the Multinational Association of Supportive Care in Cancer (MASCC) and the National Comprehensive Cancer Network (NCCN), which were both updated in 2011. Other recommendations, aimed at raising awareness of CINV and its management, include timely updates of relevant local practice guidelines and protocols, translation of the MASCC and NCCN guidelines into all European languages and their dissemination through accessible articles in nursing journals and newsletters and via nursing conferences and study days, improved training for nurses on CINV, collaboration between the European Oncology Nursing Society and national nursing organisations to promote consistent practice, the development of a CINV toolkit, information provision for patients, local audits of CINV management, and a survey of CINV management between and within European countries.

## Introduction

Chemotherapy-induced nausea and vomiting (CINV) is a recognised adverse effect of cytotoxic cancer treatment, and one that is consistently cited by patients as one of their greatest fears before embarking on a treatment course [1]. As well as having a deleterious effect on quality of life, CINV can also cause physiological impairment, loss of functional ability and a decline in performance status [2–4]. When CINV is severe, it may lead to a clinical decision to cease chemotherapy [2] or, in our clinical experience, to implement dose delays or reductions. Patients who experience ongoing CINV may decide to withdraw from further chemotherapy [2].

The risk of chemotherapy dose delay, reduction or cessation is a serious cause for concern, particularly when a course of chemotherapy has been prescribed with curative intent. It has been reported that patients with haematological malignancies who receive less than 90% of their planned chemotherapy dose intensity (total dose divided by total treatment time) are at a significant survival disadvantage compared with those who receive ≥90% dose intensity [5–7]. A relationship between survival and the proportion of the planned dose intensity that patients receive has been identified in other settings, including breast cancer [8]. In addition to the patient burden, poorly controlled CINV makes demands on healthcare resources [1].

Around 70–80% of patients receiving chemotherapy are at risk of CINV [2], and various factors affect the extent of the risk. For example, chemotherapy regimens vary in their emetogenicity, depending on the agent/agents used and their dosage. A widely used classification system is based on the frequency of emesis associated with a given agent when used without effective CINV prophylaxis. For example, highly emetogenic chemotherapy (HEC) denotes a CINV risk of >90% and moderately emetogenic chemotherapy (MEC) denotes a CINV risk of 31–90% [1,2]. Various patient factors are also known to affect the likelihood of CINV. For example, the risk is higher in women than in men, in younger (<50 years) than in older patients and in those with a history of motion sickness, and it is reduced in patients with a high alcohol consumption [1,2]. Previous experience of CINV is another risk factor (see anticipatory CINV, below).

CINV is categorized according to the timing of its occurrence relative to the administration of chemotherapy. Acute CINV describes nausea or vomiting that occurs during the 24 hours following a dose of chemotherapy; it generally reaches a peak of intensity after 5–6 hours [2]. Delayed CINV refers to nausea or vomiting that begins at least 24 hours following the dose of chemotherapy. For example, the intensity of CINV in patients receiving treatment with cisplatin-based chemotherapy (classified as HEC) may be at its highest 48–72 hours after treatment and can last for up to a week [2]. Some patients who have experienced nausea or vomiting after a previous cycle of chemotherapy develop further episodes prior to subsequent doses, i.e. anticipatory CINV [2]. In the experience of the authors, patients may suffer anticipatory nausea and/or vomiting as soon as they arrive in the chemotherapy clinic or at any other stage up to and including the initiation of the infusion. Breakthrough CINV describes nausea and/or vomiting that occurs despite the use of CINV prophylaxis and requires active management with rescue medication [2].

CINV management has become a fertile area for pharmacological research, resulting in a range of antiemetic interventions undreamt of 20 years ago, and the development of evidence-based treatment strategies supported by international guidelines [2,9,10]. The cornerstone of current recommendations for CINV management is effective prophylaxis, i.e. treatment given before the patient develops the first symptoms of nausea or vomiting. The latest international guidelines—2011 updates to recommendations from the Multinational Association of Supportive Care in Cancer (MASCC) [10] and the National Comprehensive Cancer Network (NCCN) [2]—set out a multi-drug approach to CINV prophylaxis for patients receiving HEC and MEC regimens (Tables 1 and 2). The key drug classes are 5-hydroxytryptamine type 3 receptor antagonists (5HT_3_ RAs), neurokinin-1 receptor antagonists (NK-1 RAs) and corticosteroids. Within these classes, MASCC specifically recommends the NK-1 RA aprepitant for both HEC and MEC and, in its MEC guidelines, it specifies the 5HT_3_ RA palonosetron on day 1 of chemotherapy. Both 2011 guidelines indicate dexamethasone as the corticosteroid of choice.

Despite the recent updates to the guidelines and their ready availability on the internet, there are anecdotal reports that the recommendations are not fully implemented, leading to inequalities of care and, potentially, suboptimal treatment outcomes for some patients. In a bid to share our experiences of CINV management in light of the latest guidelines, a group of seven senior nurses with expertise in cancer care from five western European countries (France, Germany, Portugal, Spain and the UK) met in London in June 2010 to form a European CINV Forum.

This article outlines the discussions of the European CINV Forum, and our recommendations for optimal management of CINV.

## Implementation of CINV recommendations: shared experience

### Awareness of guidelines

Although we, the members of the European CINV Forum, believe that most cancer physicians are aware of the CINV guidelines, our discussions revealed that some doctors in Germany, Portugal, Spain and the UK prefer to stick with their own way of managing CINV. In Spain, for example, there is often insufficient consideration of the optimal use of antiemetic drugs and of the dosage and timing of such treatment. In the UK, there is variability in the implementation of antiemetic guidelines, particularly regarding prophylactic use of aprepitant and palonosetron, and a tendency to use a one-size-fits-all approach based solely on the regimen-based risk and not the risk associated with individual patient factors.

There is low awareness of the guidelines among nurses in Germany and France. In general, we reached the conclusion that nurses are unlikely to read guidelines or other pertinent documents unless they are available in the local language and well publicized in appropriate media.

### Local practice guidelines

Local guidelines often determine which CINV drugs are available to patients. In Portugal, although there tends to be a delay in implementing international guidelines, once local guidelines are in place hospitals do try to prescribe accordingly. The two UK Forum members noted that local NHS guidelines generally lag behind international recommendations—sometimes by several years. For privately insured patients in the UK there is no such delay, since access to licensed drugs is based on clinical efficacy and physician choice, and not dictated by cost. It is, therefore, potentially easier to implement international guidelines in the private healthcare sector.

### Impact of funding issues

We reached the conclusion, through our discussions, that healthcare funding appears to be impeding the implementation of CINV guidelines in some countries. For example, in Portugal, cancer care has a relatively low national priority and some cancer centres do not have access to the full range of modern antiemetic treatments. In Spain, the funding of intravenous antiemetic treatment is largely hospital-based, while oral treatment is generally funded via primary care or the community. In the UK, doctors who readily prescribe guidelines-based CINV prophylaxis for their private patients are often unable to apply the same standard of care in their National Health Service practice because of restrictions imposed by local funding decisions. In Germany, although the availability of chemotherapy is decided at national level, supportive care is paid for via patients’ health insurance, and there is variation in the terms of these policies. In Germany, pharmacists may intervene to change the patient’s CINV treatment, e.g. advising a switch to an alternative (probably cheaper) drug. In Portugal, doctors may change the protocol of CINV management so that an alterative (sometimes cheaper) alternative is used.

France does not face particular difficulty with the funding of CINV treatments, largely because the drugs are funded by community health-care services rather than by the acute sector.

Where access to optimal CINV management is poor, we believe there is lack of awareness of the financial burden associated with poorly controlled nausea and vomiting, i.e. the costs of hospital admission for intravenous rehydration in severe CINV, and also the costs for patients in terms of loss of function and productivity.

### Nursing roles

There is some variation between the countries in the influence of nurses on the healthcare agenda. For example, where the UK has specialist nurses, nurse prescribers and an infrastructure of academic nursing, none of these cultures exists in France. The German Forum member reported a tendency for nurses not to question decisions made by their medical colleagues. As a whole, we felt that nurses—even those with specialist knowledge and experience of supportive care for patients receiving chemotherapy—are generally not involved in the drawing up of CINV guidelines at local level.

### Patient factors

We noted that, because chemotherapy is increasingly administered in an outpatient setting, CINV is more likely to develop after the patient has gone home, rather than in the clinic. At their next assessment, many patients fail to report episodes of CINV, or understate its severity. There are various reasons why this might be the case. Some patients regard CINV as a marker of chemotherapy efficacy. Some fear that the development of adverse events such as CINV will lead to chemotherapy dose reduction, delay or cancellation. Some, when they attend for a subsequent cycle of chemotherapy, simply forget how severely CINV affected their activities of daily living, e.g. work and childcare responsibilities.

## Recommendations for optimal CINV management

The European CINV Forum makes the following recommendations:

1. All patients receiving HEC or MEC should undergo a full assessment of their risk of CINV, taking account of the emetogenicity of the planned chemotherapy regimen and patient factors such as sex, age and history of nausea and vomiting, and then the appropriate prophylactic treatment indicated in the 2011 MASCC or NCCN guidelines should be implemented [2,10].
  - An emetogenicity calculation tool, incorporating both regimen-specific and patient-specific factors, would facilitate CINV prediction.
2. As new chemotherapy agents and regimens (including dose changes) are introduced, it is imperative to establish their emetogenicity.
3. Local CINV guidelines and protocols need to be updated, in a timely way, to take account of changes to evidence-based international recommendations.
4. Nurses across Europe working with patients undergoing chemotherapy should have a clear understanding of CINV and the latest international guidelines for its management, which will require:
  - Timely availability of guidelines documents in all European languages
  - Easily accessible reviews and summaries of the guidelines in nursing journals (in local languages) and local hospital newsletters
  - Presentation of the guidelines at local, regional and national nursing conferences and study days
  - Improved training for nurses on CINV, including recognition of HEC and MEC regimens, the role of patient-related factors and the importance of optimal prophylactic treatment
  - Collaboration between the European Oncology Nursing Society and national nursing organizations to promote consistent practice in CINV management across Europe.
5. The development of a chemotherapy toxicity toolkit would improve the supportive care offered across a range of side effects; its features would include:
  - Patient diaries
  - Ready access to telephone support
  - Regular updates as new chemotherapy regimens and supportive care guidelines are introduced.
6. Patients should be offered information on CINV and its management in an attractive, reader-friendly form, emphasizing optimal management and offering practical tips on nutrition and meal preparation to help combat nausea.
7. Local audits of CINV management are needed to help healthcare practitioners understand how their policies and practices affect patient care.
8. A survey of CINV management at local level between and within European countries would provide a snapshot of current practices and a focus for change.

## Conclusion

The inaugural meeting of the European CINV Forum has provided insight into areas of both commonality and difference between the countries represented, and generated a range of recommendations for improving the management of CINV. First and foremost among these recommendations is that the CINV risk for every patient should be assessed before chemotherapy is administered, and appropriate prophylactic measures should be taken, based on the latest guidelines.

It is clear that the implementation of our recommendations must take account of the healthcare and professional cultures in each country. However, we believe that the formation of the Forum and our commitment to ongoing discussion and activity will help to improve cross-border communication of knowledge, ideas and good practice, and raise the profile of an evidence-based approach to CINV management.

## Figures and Tables

**Table 1:** CINV prophylaxis guidelines for patients receiving HEC regimens [2,10]

**Guideline**	**Drug combination on day of chemotherapy**	**Drug combination on day 2+ after chemotherapy**
MASCC	Aprepitant + 5HT_3_ RA + dexamethasone	Dexamethasone + aprepitant
NCCN	NK-1 RA + 5HT_3_ RA + dexamethasone ± lorazepam ± H_2_ blocker or proton pump inhibitor	NK-1 RA + dexamethasone ± lorazepam ± H_2_ blocker or proton pump inhibitor

MASCC, Multinational Association of Supportive Care in Cancer; NCCN, National Comprehensive Cancer Network; 5HT_3_ RA, 5-hydroxytryptamine type 3 receptor antagonists; NK-1 RA, neurokinin-1 receptor antagonists.

**Table 2:** CINV prophylaxis guidelines for patients receiving MEC regimens [2,10]

**Guideline**	**Drug combination on day of chemotherapy**	**Drug combination on day 2+ after chemotherapy**
MASCC	Palonosetron + dexamethasone	Dexamethasone
NCCN	5HT_3_ RA + dexamethasone ± NK-1 RA in selected patients ± lorazepam ± H_2_ blocker or proton pump inhibitor	5HT_3_ RA (unless NK-1 RA used on day 1) or dexamethasone or NK-1 RA in selected patients ± lorazepam ± H_2_ blocker or proton pump inhibitor

MASCC, Multinational Association of Supportive Care in Cancer; NCCN, National Comprehensive Cancer Network; 5HT_3_ RA, 5-hydroxytryptamine type 3 receptor antagonists; NK-1 RA, neurokinin-1 receptor antagonists.
